# Development of the time course for processing conflict: an event-related potentials study with 4 year olds and adults

**DOI:** 10.1186/1471-2202-5-39

**Published:** 2004-10-22

**Authors:** M Rosario Rueda, Michael I Posner, Mary K Rothbart, Clintin P Davis-Stober

**Affiliations:** 1 Psychology Dept. University of Oregon, Eugene, USA; 2 Sackler Institute, Weill Medical College, Cornell University, NY, USA

## Abstract

**Background:**

Tasks involving conflict are widely used to study executive attention. In the flanker task, a target stimulus is surrounded by distracting information that can be congruent or incongruent with the correct response. Developmental differences in the time course of brain activations involved in conflict processing were examined for 22 four year old children and 18 adults. Subjects performed a child-friendly flanker task while their brain activity was registered using a high-density electroencephalography system.

**Results:**

General differences were found in the amplitude and time course of event-related potentials (ERPs) between children and adults that are consistent with their differences in reaction time. In addition, the congruency of flankers affected both the amplitude and latency of some of the ERP components. These effects were delayed and sustained for longer periods of time in the children compared to the adults.

**Conclusions:**

These differences constitute neural correlates of children's greater difficulty in monitoring and resolving conflict in this and similar tasks.

## Background

Conflict tasks involve the selection of a sub-dominant object or response in the presence of a competing dominant object or response. One of the most common tasks used in the literature to measure conflict is the flanker task. In this task, a target surrounded by stimuli suggesting either the same (congruent) or the opposite (incongruent) response is presented. Conflict is induced by incongruent flankers which, compared to congruent ones, produce larger reaction times and reduced response accuracy [1].

Different cognitive operations appear to be involved in processing conflict [2]. First, conflict has to be detected. This involves not only the recognition of the presence of conflict in the display but also the evaluation of the degree of conflict and the realization that the situation calls for a particularly careful action, therefore we use the term *conflict monitoring *to describe these operations. Once conflict is detected, it is necessary to determine the appropriate action in a goal-directed manner. Depending on the task, the resolution of conflict might involve different processes (e.g. inhibition, rule-holding, set switching, planning, etc.).

Monitoring and resolving conflict is a function of executive attention [3]. A network of brain areas have been shown to be active in tasks that involve conflict between stimulus dimensions, including the Stroop, flanker and spatial conflict tasks. These three tasks have been shown to activate a common neural network including the anterior cingulate cortex (ACC) and lateral prefrontal areas [2].

Recent studies have dissociated the brain areas within the executive network that are responsible for monitoring and resolving of conflict [4]. In a fMRI study, [5] the ACC was shown to be involved in the detection and monitoring of conflict, while lateral prefrontal areas have been shown to be mainly related to processes required to resolve the conflict [6]. Conflict processing has also been anatomically dissociated from orienting to relevant information that involves areas of the superior parietal cortex and superior frontal gyrus [7].

Young children have more difficulty than older children and adults performing tasks that involve conflict. Using conflict tasks adapted to children, we have reported a considerable reduction in the amount of interference produced by distracting information in children from 2 to 3 years of age [8]. This reduction continues up to 7 years but we have found a striking stability after this age up to adulthood [9]. We have interpreted these data as indicating a greater difficulty in monitoring and resolving conflict from competing stimulation in young children compared to older children and adults. The greater susceptibility to interference from irrelevant stimulation for young children has been reported using many different tasks: Flanker [10-12], S-R compatibility [13], Stroop [14], negative priming [15], etc. Other tasks have been used to assess the ability to inhibit non-appropriated responses (e.g. Go-NoGo [6] and Stop-signal [16,17] tasks). In these studies, children also show greater difficulty than adults in controlling prepotent, but incorrect, responses. Depending on the difficulty of the task, developmental differences in the ability to resolve conflict between children and adults can be observed up to middle childhood and early adolescence, suggesting that full maturation of the executive control network does not take place until early adulthood.

Some developmental studies have been carried out using neuroimaging aimed at understanding the brain mechanisms that underlie the development of executive functions. For instance, Casey and her colleagues [6] conducted a fMRI study comparing 7 to 12 year old children and adults in a Go-NoGo task. Despite a very similar pattern of activations in the prefrontal cortex following No-Go trials, the average volume of activation was significantly greater for children than for adults. The same pattern of results was obtained in similar studies [18,13], suggesting that the brain circuitry underlying executive functions is more focal and refined as it becomes more efficient with development. However, due to the limited time resolution of MRI, these studies cannot analyze whether there are additional maturational differences in the time course of activation of these areas.

A number of studies have used the high temporal resolution of event related potentials (ERPs) to assay the timing of action-monitoring processes with adults. The N2 is one of the ERP indexes that have been associated with executive attention. The N2 is a pre-response negative deflection in the ERP at around 300 ms post-stimulus, which appears to be larger (more negative) for trials that involve more conflict. The N2 is observed over parietal and frontal leads and has been obtained with both flanker [19,20] and Go-NoGo tasks [21]. In both situations, the N2 has been associated with the withholding of a prepotent, but inappropriate, response. In a recent ERP study with a flanker task, van Veen and Carter [20] linked the scalp distribution of activity associated with the N2 to a source of activation originating at the caudal portion of the ACC, supporting a connection between this electrophysiological index and the executive attention network.

Only a few ERP studies have been conducted with children using conflict tasks. In one of these studies, a flanker task was used to compare conflict resolution in three groups of children aged 5 to 6, 7 to 9 and 10 to 12, and a group of adults [12]. As expected, the behavioral results showed a consistent reduction of the interference produced by the flanker with age. In addition, developmental differences were found in two ERP components, the lateralized readiness potential (LRP) and the P3. The LRP seems to be related to response preparation [22] while P3 is thought to be an index of stimulus evaluation [23]. Ridderinkhof & van der Molen [12] found differences between children and adults in the latency of the LRP, but not in the latency of the P3 peak, suggesting that developmental differences in the ability to resist interference are mainly related to response competition and inhibition, but not to stimulus evaluation.

Recently, Davis et al. [24] conducted an ERP study using a Go-NoGo task with a group of 6 year old children and a group of adults. In this study, differences between children and adults in the latency of the P3 peak were also reported. Both the amplitude and the latency of the P3 were greater for NoGo trials compared to Go trials, although this pattern was similar for children and adults. Nevertheless, in contrast with the literature, no differences in the amplitude of the N2 component were observed as a function of type of trial. Finally, a late positive component (LPC) was observed only for children over the frontal leads. The amplitude of this component was reduced for NoGo trials compared to Go trials. This difference started around 550 ms post stimulus and extended over a time window of 600 ms. The modulation of the amplitude of the LPC might result from the greater prefrontal activation observed in children when a response has to be inhibited, as shown by imaging studies that used the same type of task [6]. This would be consistent with the study by Ridderinkhof and van der Molen, in which developmental differences seem to appear in ERP components related to response selection to a greater extent than those associated with stimulus selection.

So far, the literature suggests that monitoring and resolution of conflict involve separate brain areas in adults, and that children activate similar, but somewhat larger areas. Moreover, the N2 component of the ERPs appears to be related to activation coming from the ACC and to be mainly associated with conflict monitoring, whereas later components (e.g. LPC) might result from prefrontal sources of activation and could be related to conflict resolution.

**Figure 1:**
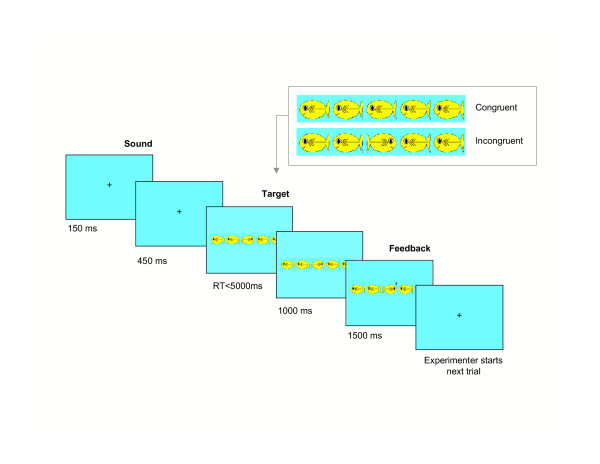
Representation of the flanker task used in the experiment

The flanker task is an appropriate experimental paradigm for assessing conflict processing. The aim of this study was to use a version of this task with children and adults to assess developmental differences in the time course of the different operations involved in conflict processing. We have recently developed a flanker task appropriated for use with children as young as 4 years [9]. In this task, a row of five fish appear in the center of the screen and the child's job is to help "feed" the middle fish by pressing the key that corresponds to the direction in which the middle fish is pointing. In the congruent trials, the fish surrounding the middle one point in the same direction as the middle fish, while in the incongruent trials, the flanker fish point in the opposite direction, suggesting an incorrect response (see Figure 1).

To study the time course of conflict processing, we examined the latency to significant differences between the ERPs for congruent and incongruent trials, and the sustainability over time of these differences. In the adult literature, the amplitude of the N2 component has been shown to be modulated by the congruency of distracting information in flanker tasks and has been related to conflict monitoring, but this component was not assessed in the study by Ridderinkhof and van der Molen. We aim to replicate the effect with adults using our child-friendly flanker task as well as analyzing this ERP component in 4 year olds. While there may be subcomponents of the N2 sensitive to other types of manipulations such as the degree to which the predicted identity of a display is violated [25], in our study we will focus on the effect of the congruency of flankers that are equally expected to ensure the activity we measure is related to conflict. In addition, differences between children and adults in stimulus selection processes as reflected by the P3 component could be playing a role in selecting a relevant stimulus among distractors, and these were examined. Finally, we explored whether the reduced amplitude of the LPC for NoGo situations reported by Davis et al. [24] for children, but not adults, is also observed with a flanker task. This outcome will rule out the possibility that the reduction of the LPC is associated with the withholding of a motor response, and will make more plausible the hypothesis of it being related to resolving situations that call for particularly careful actions as those in which conflict is induced by distracting flankers.

## Results

### Behavioral results

Means of the median RT and percentage of errors are shown in Table 1 for both children and adults. A mixed-designed ANOVA was performed with Group as a between subject factor and Flanker Type as a repeated measure with RT as the dependent measure. The ANOVA revealed significant main effects of Group (F(1,38) = 74.42; p < .001) and Flanker Type (F(1,38) = 6.15; p < .05) as well as a significant Group × Flanker Type interaction (F(1,38) = 4.65; p < .05). In addition, conflict effects were examined in the two groups. Conflict effects refer to the difference between congruent and incongruent conditions, and they can be measured using both RT and accuracy variables. Statistical significance of these effects was tested for each group independently using paired t-tests. The conflict effect was significant for both RT and accuracy for adults (t(17) = -6.2; p < .001 and t(17) = -2.54; p < .05 respectively) as well as for children (t(21) = -2.57; p < .05 and t(21) = -2.51; p < .05 respectively for RT and accuracy effects). Conflict effects were also examined for the subgroup of children (n = 14) with useful ERP data. For this group, the conflict RT was marginally significant (t(13) = -2.004; p = .066) while the conflict accuracy was significant (t(13) = -2.4; p < .05).

A similar 2 (Group) × 2 (Flanker Type) ANOVA was conducted using percentage of errors as the dependent measure. Again, the main effects of Group (F(1,38) = 22.33; p < .001) and Flanker Type (F(1,38) = 7.08; p < .05) were significant, whereas the Group × Flanker Type interaction was marginal (F(1,38) = 3.28; p = .078).

### ERP snalysis

**Figure 2:**
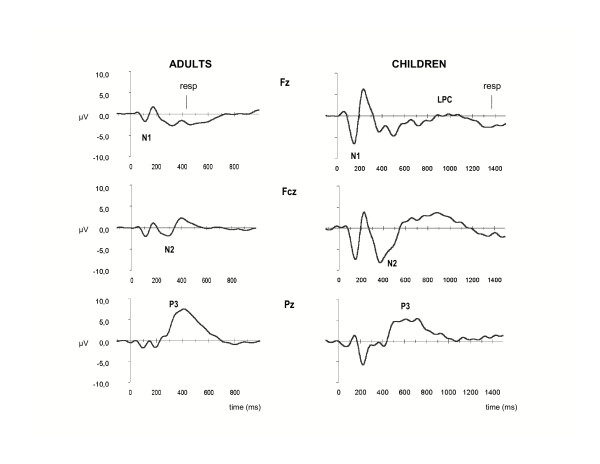
**Comparison of children's and adults' ERPs. **ERPs are the average of the artifact-free segments for correct responses.

Figure 2 shows the ERPs of adults and children at leads located at the midline of frontal and parietal sites. Despite general differences in overall amplitude and latency, the waveforms for the two groups were strikingly similar. We observed N1 and N2 components over frontal leads and a P3 over parietal leads for both children and adults. In addition, children showed a pre-response late positive component (LPC) over frontal channels.

We hypothesized that particular ERP components would be sensitive to the presence of conflict in the display. To examine these predicted effects, we computed the latency and amplitude of the peaks of the N1, N2 and P3 components in both groups and of the LPC in the group of children separately for congruent and incongruent conditions in a selection of frontal and parietal leads (see Table 2). The selected leads had equivalent locations in the children's 128 and adults' 256 channels arrays and corresponded to particular 10-10 international system positions [26]. Overall amplitude refers to the maximum negative or positive voltage values (in microvolts, μVolts) within the ERP component. Latency was computed in milliseconds (ms) from the time the target was presented to the time of the maximum positive or negative peak within the ERP component. To calculate both peak amplitudes and peak latencies we selected time-windows in which the waveforms deflections defining each ERP component were included, and computed the latency and amplitude of the peak within those windows using the tool provided by the Net Station 3.0 (EGI software). These time-windows, specified in Table 2, were different for children and adults.

Because of the greater presence of artifacts in the children's ERPs, a significantly larger number of segments were used to compute the averaged ERPs in the adult data (see Method section). To control for possible influence of this difference on the amplitude of the ERPs [27], the number of segments used to compute the averaged ERPs was included as a covariate of the differences between children and adults in the dependent measures entered in the analysis. Separate 2 (Group) × 2 (Flanker Type) × 3 (Channel) ANCOVAs with the means of peak latency and amplitude as dependent measures were conducted separately for the N1, N2 and P3 components. In addition, we conducted a 2 (Flanker Type) × 3 (Channel) ANOVA with both the peak latency and amplitude of the LPC only for children. In these analysis we used the Huynh-Feldt correction for sphericity as needed. The results of these ANOVAs are summarized in Table 3.

For the peak latency, the main effect of Group was significant in all the ERP components. The main effect of Flanker Type was significant for the N1, P3 and LPC. No significant interactions were found for any of the ERP components for the latency data.

For the peak amplitude values, the main effect of Group was significant for the N1 and N2. The main effect of Flanker Type was not significant for any of the components although it was marginally significant for the N1 and for the LPC in the children. The main effect of Channel was marginal for the N2 and highly significant for the P3. Interestingly, there was a significant Group × Flanker interaction for the P3, indicating a significant effect of the type of flankers in the peak amplitude of this component for children (F(1,30) = 6.0; p < .05) but not for adults (F < 1).

**Figure 3:**
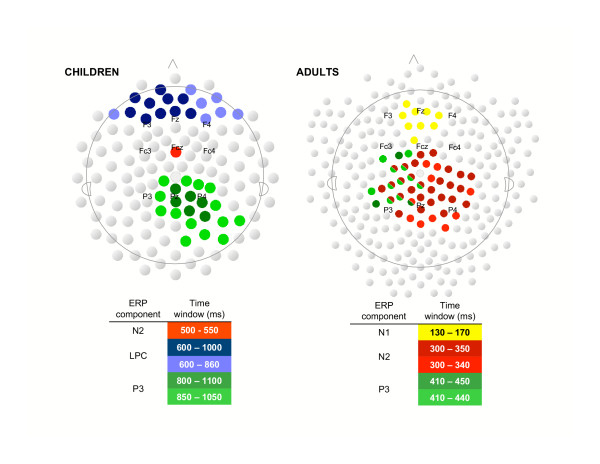
**Distribution of significant congruency effects in children and adults **Each image represents the distribution of the 128 (children) or 256 (adults) channels on the scalp. Marked channels showed significant (p < .05) congruent *vs*. incongruent differences. The time windows of the differences are color-coded and exposed in the tables below the montages.

**Figure 4:**
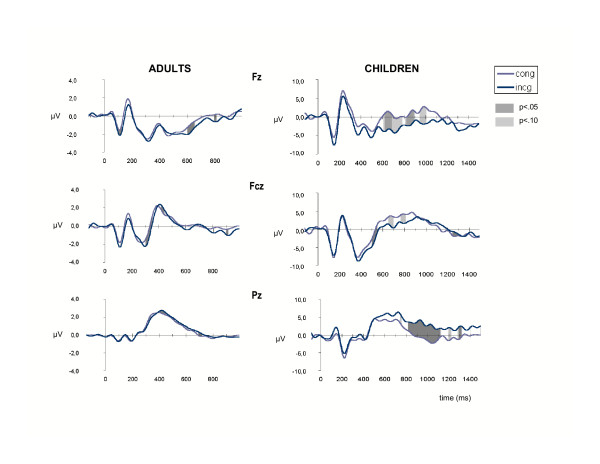
**Comparison of flanker effects in adults and children ERPs. **ERPs are the average of the artifact-free segments for correct response for each flanker condition. *Cong*: congruent trials; *Incg*: Incongruent trials.

Although the peak amplitude of ERP components is a widely used measure to look at effects of the variables of interest in the patterns of brain activation, these effects can certainly occur along the entire epoch and not only in the peaks of the components. To examine the effect of congruency in the amplitude of the registered activity in the entire epoch, we computed amplitude differences between congruent and incongruent conditions sample by sample along the ERP segment in all channels for children, and a selection of channels around the Fz, Fcz and Pz positions in the adult data. T-tests were carried out to assess the significance of these differences along the epoch. In Figure 3, the leads in which the congruent vs incongruent differences in amplitude were found significant are highlighted for both children and adults, as well as the time windows for these differences and the ERP components in which the differences appear. In addition, graphs displaying the ERPs for each flanker condition at Fz, Fcz and Pz positions are shown in Figure 4 for adults and children. The shadowed areas in these figures show the sections of the ERPs in which congruent vs. incongruent differences were significant.

#### Correlations between reaction time and electrophysiological measures

In order to explore possible associations between the amplitude and latency dimensions of the ERP components and the particular cognitive processes measured by reaction time (RT), we examined correlations between RT measures and patterns of brain activity at Fz, Fcz and Pz positions and their left and right equivalents. Correlations were computed independently for adults and children. The first set of correlations involved the conflict effect as measured by subtracting the RT for congruent trials from the RT for incongruent trials (conflict score) and the overall RT as behavioral measures, and the overall (across flanker conditions) latency and amplitude of the ERP components as electrophysiological measures. For the adults, the overall RT correlated negatively with the amplitude of the N1 at channel F3 (r = -0.53; p < .05), and positively with the latency of the N2 component at channel Fc4 (r = 0.47; p < .05). For the children, the overall RT correlated negatively with the amplitude of the P3 at channel P4 (r = 0.72; p < .01), and positively with the latency of the N1 at channel F4 (r = 0.60; p < .05) and the N2 at channel Fcz (r = 0.58; p < .05). No significant correlations were established between any of the overall ERP components and the conflict score in either adults nor children.

Finally, correlations were calculated between the behavioral measures of overall RT and conflict score, on the one hand, and the effect of flankers on the amplitude and latency of the peaks of the ERP components on the other. For the adults, overall RT correlated positively with the N2 latency effect at Fcz (r = 0.59; p < .01) and the P3 amplitude effect at P4 (r = 0.46; p = .05), whereas the correlation was negative with the N2 latency effect at Fc4 (r = -0.54; p < .05). On the other hand, the conflict score correlated positively with the amplitude effect on the N2 at Fc4 (r = 0.47; p < .05) and Fcz (r = 0.41; p = .09), although the last effect was only marginal. In the children, the overall RT correlated negatively with the N2 latency effect at Fc3 (r = -0.56; p < .05), and marginally with the N1 amplitude effect at Fz and F4 (r = -0.50; p = .07 and r = -0.46; p = .09 respectively). However, the conflict score correlated negatively with the P3 latency effect at channel P4 (r = -0.81; p < .001).

## Discussion

As expected, young children showed increased difficulty compared to adults in both processing the target and dealing with distracting information incongruent with the correct response. The greater difficulty of the task for children was reflected in children's much longer overall RT and conflict scores. The main goal of the current study was to analyze the differences in brain activation between children and adults underpinning their behavioral differences. Our results show differences among children and adults in both the time course of brain activations overall and across flanker conditions.

### Time course of target processing

Significantly larger N1 and N2 amplitudes were found for children than for adults, whereas the P3 showed equivalent amplitudes in the two groups. Children usually show larger event related potentials and often with delayed latency compared to adults [12,24]. These differences in general amplitude and latency relate to a variety of maturational factors as brain size, skull thickening and synaptic density [28]. It is not clear how the amplitude of the ERPs components relates to the effort to process the target. In both adults and children, the overall RT correlated negatively with the amplitude of some of the waveform components (N1 for adults, P3 for children), consistent with the idea that the amplitudes of the ERPs components are associated with cognitive operations that can facilitate the speed of processing the target.

Differences in latency of the ERPs components can be of special interest when it comes to accounting for differences in RT. Accordingly, children showed significant delays in the latency of all components compared to adults. The difference between children and adults was greater in the later components, suggesting that children's delay in target processing is more pronounced in later stages of processing. An objection to this conclusion is that latency and amplitude of the waveforms deflections are not independent, given the fact that greater peak latencies can be expected with more pronounced differences in amplitude. However, two pieces of information in our data point to the fact that differences in amplitude cannot account for all differences in latency. First, the P3 component shows a large difference in latency despite no overall differences in amplitude. Second, the overall RT appears to correlate negatively with the amplitude of some of the components in both children and adults, whereas it correlates positively with latency measures.

In addition, overall RT appear to correlate with the overall amplitude and latency of some ERP components, while no significant correlations are established between these and the conflict score, suggesting that the general speed of processing but not the ability to manage conflict might be related to the general form of the ERP.

### Time course of conflict resolution

It should be borne in mind in comparing the adult data with previous studies conducted with other flanker tasks that the child-friendly version of this task used in our study was very easy for adults. This could account for the modest amplitude differences between congruent and incongruent trials in this study compared to what has been found with other versions of the flanker task [29]. From when children are first able to perform reaction time tasks, the time to respond appears to decline linearly to adulthood as do the conflict scores up to seven years of age [9]. The continuous nature of the two behavioural reductions suggest that although the flanker task might be easier for older children and adults the same mental processes are involved.

As shown in Table 2, the manipulation of congruence between relevant and distracting information in the display produced some effects on both the amplitude and latency of the ERP elicited by the target (see also Figure 4). In consonance with the literature, adults show an effect in the N2 component at frontal and parietal areas around the midline as well as an effect on the P3 observed at the left and mid parietal leads. For the P3, these effects are found on the latency of the peaks as well as the amplitude of particular time windows within the components (see Figure 3). For the N2, the effect is mainly observed on the amplitude of the component. In addition, adults show an effect on the amplitude and latency of the N1 that is localized at the frontal midline (Fz).

On the other hand, 4 year old children do not show differences in brain activity among the two flanker conditions until approximately 500 ms post target. Therefore, the effect of flankers is not observed at this age in the relatively early N1 component, and only very weakly at the N2 (see Figures 3 &4). However, as in the adults, children show robust frontal and parietal effects. The frontal effect is observed in the LPC, and consists of a less positive amplitude of the component during incongruent relative to congruent trials. The parietal effect in children is observed in a late P3 component that, as for adults, consists of a greater amplitude for incongruent trials than congruent ones. Although the frontal effect appears around 200 ms earlier than the parietal effect, in both cases, the amplitude effect lasts for over 500 ms. The amount of time the amplitude difference is sustained constitutes an important difference between children and adults, and may reflect the time course of brain mechanisms supporting the monitoring and resolution of conflict.

As mentioned in the introduction, the N2 effect has been consistently found in different versions of the flanker task, and has been related to action-monitoring processes implemented in the anterior cingulate [20]. Botvinick, et al [5] more precisely specified the role of ACC in detecting and signaling conflict. In consonance with these data, our results showed a modest positive correlation between the effect of flankers on the amplitude of the N2 and the conflict score in adults.

It is less clear what type of cognitive operation is underlying the P3 effect. In a review of mental chronometry, Coles et al. [30] distinguished between amplitude and latency effects in their analysis of the conditions that elicit the P3. According to these authors, amplitude differences in the P3 can be elicited by stimuli that differ in their probability (either objective or subjective) of occurrence, but also in the amount of goal-relevant information contained in the stimulus, whereas latency differences might be associated with time differences in stimulus evaluation or categorization. In our task, the two types of trials had equal probability of occurrence. Consequently, the greater P3 amplitude for incongruent trials is more likely to be associated with the need for a more careful evaluation of the stimulus to determine the correct response. If we only look at the amplitude effect on the P3 at the particular time window in which the effect is found significant in the adult data, a positive correlation between the amplitude effect on the P3 and the conflict score is found (r = 0.62; p < .01). This suggests that the greater the effort to select the correct response, the greater the relative P3 amplitude for incongruent trials, and therefore the greater the conflict score.

Our data fit quite well within the sequence of cognitive operations suggested by the literature. In the adults, conflict detection, as reflected by the frontal effects, is a few tens of milliseconds delayed for incongruent trials. Immediately after, we observe an effect over parietal leads apparently related to the effort to determine the correct response. This process is approximately 40 ms delayed when incongruent flankers are presented. Nonetheless, the delays in the N2 and P3 components under incongruent conditions may not be completely independent, as suggested by their quite overlapping topographies (see Figure 3).

In the children, we have also observed frontal and parietal effects occurring prior to the response. However, probably due to their generally slower capacity for processing information, the frontal effect is quite delayed in comparison with adults, and mostly observed on the LPC instead of the N2. Although determining whether the frontal effects observed in these two different ERP components in children and adults are equivalent will require further research, the fact that the effect on the LPC occurs over the frontal leads and prior to the parietal effect supports its involvement in conflict monitoring.

Likewise, a remarkable increase in the amplitude and delay of the latency of the P3 peak is observed for the children on incongruent trials. In consonance with the result of the study by Ridderinkhof & van der Molen [12], both children and adults showed the effect on the peak latency to the same degree. However, in their study, Ridderinkhof & van der Molen did not report amplitude effects. Interestingly, our data reveal a greater effect on the P3 amplitude for the 4 year old children compared to the adults. This suggests that children at this age take longer than adults in evaluating the display. This delay occurs in addition to the delay in response selection revealed by the adults vs. children differences in the LRP shown by Ridderinkhof & van der Molen.

At both the frontal and parietal components, the flanker effect is sustained for a longer period of time in the case of children. These differences in patterns of brain activation are likely to underlie the observed behavioral differences in conflict resolution between children and adults. Certainly, the brain processes underlying the detection of conflict and the selection of the appropriate response appear to take longer to be resolved into a correct action in the brains of 4 year old children.

The distribution of the flanker effects shown in Figure 3 appears to be another important difference between 4 year old children and adults. In adults, the frontal effects appear to be focalized in the mid line (Fz for N1, and Fcz for N2), while in children we observed the effects mostly at pre-frontal sites and in a broader number of channels, including the mid line (Fz) and leads on the left (F3) and right (F4) sites. In addition, the effect on the P3 appears to be left-lateralized in the adults data but lateralized to the right side in the children. The focalization of the signals in adults as compared to children is consistent with neuroimaging studies conducted on developmental populations in which children appear to activate a broader area of the brain compared to adults when exposed to the same task [13,18].

## Conclusions

A major new finding of this study is the difference found between 4 years old children and adults in the longer latency and the sustained congruency effect on the ERPs. Consistent with their larger conflict scores in reaction time, these differences shed light on the brain mechanisms underpinning the much greater difficulty for children in monitoring and resolving conflict.

## Methods

### Participants

Eighteen young adults (12 women, 6 men; mean age: 23 years; SD: 6.45) and twenty-two children (11 girls, 11 boys; mean age: 4 years, 4 months; SD in months: 2.2) participated in the study. All participants were right-handed. The adult participants and the parents of children involved in the study gave written consent prior to the experimental session. Both children and adults were paid for participating in the study.

### Procedure

The stimulus sequence for each trial was controlled using E-Prime (Psychological Software Tools, Pittsburgh, PA). Each trial began with a sound to alert participants about the start of the trial. One second after the sound, a line with five drawn fish was presented in the center of the screen (Figure 1). The central fish was the target, and the ones on the sides the flankers. Participants were instructed to press the mouse button that matched the direction toward which the middle fish was pointing while ignoring the flanker fish. Half of the trials were congruent and half incongruent. In the congruent trials, the five fish were pointing in the same direction; in the incongruent trials, the flanker and target fish were pointing in opposite directions. The experiment was presented to the children as a game in which they will be shown a hungry fish surrounded by other fish. The children were told the hungry fish is always the one in the middle and that they will make it happy by feeding it when they press the key corresponding to the direction it is swimming. The target display was presented until a response was made, or up to 1700 ms in the case of adults, or 5000 ms in the case of children. After the response was given, the display did not change for another second, after which feedback was provided. Feedback consisted of a 1500 ms long animation of the middle fish, showing it happy (bubbles coming up from his mouth) for the correct response, or sad (bubbles coming down the eye) for the incorrect or missed trials. The inter-trial interval was 1500 ms for adult participants. For the children, the experimenter initiated each trial once the child was focused on the computer monitor. A fixation cross was continuously displayed in the center of the screen except when targets and feedback were presented. All participants were instructed to be as fast and accurate as possible. Both children and adults completed five blocks of 20 trials each, preceded by 12 practice trials. Children could repeat the practice block as many times as needed until it was clear they understood the instructions. The experimental session was about 35 minutes long for adults and 45-to-60 minutes long for children.

### EEG recording and data processing

EEG was recorded using a 128-channel Geodesic Sensor Net [31] for children, and a 256-channel net for adult participants. The GSN is a reliable method for acquiring high-density EEG data, and given its fast application, this method is specially convenient for children [32].

The EEG signal was digitized at 250 Hz. Impedances for each channel were measured prior to recording and kept below 80 kΩ during testing. Recording in every channel was vertex-referenced and the time-constant value was 0.01 Hz for both children and adults. Data were recorded using Net Station 2.0 (EGI Software) and processed using Net Station 3.0.

Once acquired, data were filtered using a FIR bandpass filter with 12 Hz low-pass and 1 Hz high-pass cutoffs. Continuous EEG data was segmented into target-locked epochs. The epochs were 1 sec. long for adults (-200 ms to 800 ms around target) and 1.7 sec. long for children (-200 ms to 1500 ms around target). Segmented files were scanned for artifacts with the Artifact Detection NS tool using a threshold of 70 μV (adults) or 100 μV (children) for eye blinks and eye movements. Segments containing eye blinks or movements as well as segments with more than 25 bad channels were rejected. Within each segment, channels with an average amplitude of more than 200 μV or a difference average amplitude of 100 μV were also discarded from further processing. Finally, particular channels were rejected if they contained artifacts of any kind in more than 50% of the segments. Children's data were also visually inspected trial by trial to make sure the parameters of the artifact detection tool were appropriate for each child. As a consequence of the artifact detection procedure, an average of 36% of the ERP segments in the children data and an average of 18.5% of the ERP segments in the adults were rejected. The larger number of rejected segments for the children was due to a higher frequency of blinks, mouth and/or head movements, speaking, and other behaviors that generate artifacts on the EEG signal during the experimental procedure. Thus, we decided to have a criterion of a minimum of 12 clean segments per flanker condition among the correctly responded trials for further processing individual data. All adults participants and a total of 14 children reached this selection criterion. The average number of segments included in the averaged ERPs was 53.2 (SD: 23.1) for the children (26.6 per flanker condition; SD: 11.41 and 11.48 respectively for congruent and incongruent conditions), and 80.3 (SD: 17.3) for adults (40.3, SD: 17.3 for congruent trials; and 40.0, SD: 9.8 for incongruent trials), and this children *vs *adults difference was significant (t(23.4) = -3.66; p < .001).

Artifact-free segments for correct responses were averaged across conditions and subjects and re-referenced against the average of all channels. The 200 ms preceding the target served as baseline.

## Authors' contributions

MRR, MIP & MKR designed the study and participated in the theoretical elaboration of the paper. MRR was responsible for the data collection and data analysis processes. CPD designed and performed part of the statistical analysis on the EEG data.

## Figures and Tables

**Table 1:** Children and adults RT (in ms) and percentage of errors in the flanker task

	*RT*				*% Errors*			
	
	*Overall*	*C*	*I*	***Conflict***	*Overall*	*C*	*I*	***Conflict***
*Adults*	431 (86)	415 (83)	445 (85)	30** (20)	1.4 (1.9)	0.2 (0.65)	2.6 (0.65)	2.3* (3.9)
*Children (n = 22)*	1614 (489)	1490 (476)	1913 (916)	424* (773)	16.7 (13.6)	10.6 (12.4)	22.87 (21.9)	12.98* (22.9)
*Children (n = 14) *^*a*^	1385 (349)	1292 (327)	1525 (563)	**233^# ^(436)**	10.4 (8.2)	8.4 (8.9)	12.38 (8.7)	3.95* (6.16)

SDs are shown in brackets. Conflict is measured by subtracting congruent (C) from incongruent (I) data. Significance of the conflict effects (using t-tests): ** p < .001; * p < .05; ^# ^p < .07. ^a ^Children with a minimum of 12 clean ERP segments

**Table 2:** Latency and amplitude of the peak of each ERP component by channel location, flanker condition and group

		*N1*			*N2*			*P3*			*LPC*		
		
		*Fz*	*F3*	*F4*	*Fcz*	*Fc3*	*Fc4*	*Pz*	*P3*	*P4*	*Fz*	*F3*	*F4*
***Peak Latency***													
Adults	*C*	111 (13.5)	114 (15.8)	110 (17.8)	264 (42.6)	272 (47.7)	279 (47.2)	397 (55.7)	377 (60.0)	381 (59.3)			
	*I*	117 (15.6)	119 (17.3)	110 (24.2)	286 (46.3)	273 (54.0)	266 (50.6)	430 (69.4)	418 (87.8)	389 (72.4)			
Children	*C*	147 (14.4)	146 (14.4)	152 (18.9)	395 (54.8)	401 (49.8)	399 (55.7)	562 (70.9)	613 (158.5)	600 (156.9)	843 (179.6)	881 (153.5)	890 (141.7)
	*I*	151 (12.2)	155 (16.0)	154 (14.9)	391 (46.2)	405 (49.9)	397 (63.0)	654 (168.2)	691 (193.6)	622 (132.9)	1014 (180.5)	1054 (169.4)	939 (192.9)
***Peak Amplitude***													
Adults	*C*	-2.14 (1.4)	-1.92 (1.41)	-1.75 (1.3)	-3.56 (3.42)	-2.78 (1.83)	-2.69 (2.06)	8.40 (3.88)	5.82 (2.55)	7.81 (4.97)			
	*I*	-2.55 (1.32)	-2.13 (1.39)	-2.01 (1.21)	-3.70 (3.03)	-2.70 (1.55)	-2.58 (1.78)	8.49 (3.40)	5.56 (2.19)	7.37 (4.85)			
Children	*C*	-6.20 (4.59)	-6.99 (3.34)	-7.15 (3.97)	-9.59 (4.54)	-9.46 (4.03)	-8.91 (3.45)	8.92 (4.79)	6.23 (3.33)	9.38 (5.11)	6.60 (3.69)	6.33 (3.8)	5.29 (2.61)
	*I*	-8.27 (5.24)	-9.29 (5.56)	-8.29 (3.93)	-10.55 (4.84)	-9.22 (4.83)	-9.04 (3.32)	10.91 (3.53)	7.15 (3.35)	12.14 (4.95)	3.39 (3.51)	3.53 (3.51)	4.06 (2.91)

SDs are shown in brackets. The latency values are expressed in milliseconds and the amplitude values in μVolts. The time-windows used to compute the latency and amplitude values were the following respectively for adults and children: 50–150 ms and 100–200 ms for the N1, 200–400 ms and 300–550 for the N2, 250–650 ms and 400–1100 ms for the P3; and 550–1300 for the LPC in children. *C*: congruent; *I*: incongruent.

**Table 3:** Summary of results of the ANCOVAs performed for each ERP component using the peak latency and peak amplitude data

	N1		N2		P3		LPC	
	
	F	df	F	df	F	df	F	df
	
***Peak Latency***								
Group (G)	31.7***	1, 29	58.1***	1, 29	57.53***	1, 29		
Flanker (F)	4.41*	1, 30	<1	1, 30	8.09**	1, 30	8.37*	1, 13
Channel (Ch)	<1	1.5, 43.9	<1	1.5, 46.1	<1	1.8, 53.8	1.66	1.5, 19.2
G × F	<1	1, 29	<1	1, 30	1.31	1, 30		
G × Ch	3.17^#^	1.5, 43.9	<1	1.5, 46.1	1.22	1.8, 53.8		
F × Ch	2.49	1.6, 48.6	1.37	1.5, 45	1.78	2, 60	3.13^#^	2, 26
G × F × Ch	<1	1.6, 48.6	1.16	1.5, 45	<1	2, 60		
***Peak Amplitude***								
Group (G)	27.67***	1, 29	24.49***	1, 29	1.02	1, 29		
Flanker (F)	3.33^#^	1, 30	<1	1, 30	2.69	1, 30	3.54^#^	1, 13
Channel (Ch)	<1	1.7, 52.3	3.07^#^	2, 60	15.63***	1.6, 47.6	<1	2, 26
G × F	1.75	1, 30	<1	1, 30	4.13*	1, 30		
G × Ch	3.46*	1.7, 52.3	<1	2, 60	1.71	1.6, 47.6		
F × Ch	<1	2, 60	1.26	2, 60	1.31	1.6, 48.7	2.84	1.4, 18.5
G × F × Ch	<1	2, 60	<1	2, 60	1.72	1.6, 48.7		

The number of segments used to compute the averaged ERPs was included as a covariate to account for group differences. Significance of effects: *** p < .001; ** p < .01; * p < .05; ^# ^p < .10.
